# Cramping, crashing, cannulating, and clotting: a qualitative study of patients’ definitions of a “bad run” on hemodialysis

**DOI:** 10.1186/s12882-020-01726-8

**Published:** 2020-02-27

**Authors:** Pei-Yi Kuo, Rajiv Saran, Marissa Argentina, Michael Heung, Jennifer Bragg-Gresham, Sarah Krein, Brenda W. Gillespie, Kai Zheng, Tiffany C. Veinot

**Affiliations:** 1 School of information, University of Michigan, 4314 North Quad, 105 S. State St, Ann Arbor, MI 48109-1285 USA; 2 Institute of Service Science, National Tsing Hua University, HsinChu, Taiwan; 3 Division of Nephrology, Department of Medicine, University of Michigan, Ann Arbor, MI USA; 4 Kidney Epidemiology and Cost Center, University of Michigan, Ann Arbor, MI USA; 5 National Kidney Foundation, New York City, NY USA; 6 Department of Internal Medicine, University of Michigan Medical School, Ann Arbor, MI USA; 7 Veterans Affairs Ann Arbor Health Services Research & Development Center for Clinical Management Research, Ann Arbor, MI USA; 8 Department of Biostatistics, School of Public Health, University of Michigan, Ann Arbor, MI USA; 9 School of Information and Computer Sciences, University of California, Irvine, CA USA; 10 School of Public Health, University of Michigan, 4314 North Quad, 105 S. State St, Ann Arbor, MI 48109-1285 USA

**Keywords:** Hemodialysis, Complications, Qualitative, Patient perspectives, Patient safety

## Abstract

**Background:**

Hemodialysis sessions frequently become unstable from complications such as intradialytic hypotension and untoward symptoms. Previous patient safety initiatives promote prevention of treatment complications; yet, they have placed little specific focus on avoidable session instability. A patient-centered definition of session instability grounded in patient experiences, and an understanding of patient perceptions of causes and solutions to instability, may enable such efforts.

**Methods:**

Twenty-five participants participated in three focus groups and/or a survey. They were purposively sampled for variation in region of residence, and sensitivity to patient well-being. Focus group recordings were analyzed using descriptive coding, in vivo coding, and thematic analysis.

**Results:**

Patients define unstable sessions (“bad runs”) as those in which they experience severe discomfort or unanticipated events that interfere with their ability to receive therapy. Bad runs were characterized primarily by cramping, low blood pressure (“crashing”), cannulation-related difficulties (“bad sticks”), and clotting of the dialysis circuit or vascular access. Patients believed that cramping and crashing could be explained by both patient and clinician behavior: patient fluid consumption and providers’ fluid removal goals. Patients felt that the responsibility for cannulation-related problems lay with dialysis staff, and they asked for different staff or self-cannulated as solutions. Clotting was viewed as an idiosyncratic issue with one’s body, and perceived solutions were clinician-driven. Patients expressed concern about “bad runs” on their ability to achieve fluid balance.

**Conclusions:**

Findings point to novel priorities for efforts to enhance hemodialysis session stability, and areas in which patients can be supported to become involved in such efforts.

## Introduction

Patients receiving hemodialysis often experience low health-related quality of life (HRQOL), including pain, fatigue, and emotional distress [1]. HRQOL is so important to patients that a recent study showed that 94% of hemodialysis patients surveyed would undergo daily hemodialysis in return for an improvement in HRQOL, but only 19% would undergo this treatment frequency for an increase in survival [2].

Patient symptoms, some of which occur during hemodialysis, contribute to low HRQOL [3, 4]. Patients report severe fatigue in 50% of hemodialysis sessions, and cramping in 30% [5]. Additionally, an average of 20% of hemodialysis sessions involve intradialytic hypotension (IDH) (a systolic blood pressure that falls below 100 mmHg) [6–9]. IDH can result in symptoms such as cramping, dizziness, nausea, vomiting, syncope, and fatigue [10]. Repetitive IDH is associated with cumulative cardiovascular and other organ system injury [11–13], including myocardial stunning [14, 15]. Such intradialytic occurrences can be considered complications, which are defined here as untoward events or problems that occur during or as a result of treatment, therapies or procedures, and which may be expected or unexpected, and iatrogenic or non-iatrogenic. While some intradialytic complications are unavoidable, others may be preventable with proactive measures. As in other medical fields such as surgery [16] and critical care [17], avoidable complications of hemodialysis are identifiable as a patient safety issue [18]. In this paper, we define patient safety as the prevention of hemodialysis-related complications.

Hemodialysis-focused patient safety initiatives have mainly focused on important problems such as medication errors, fall prevention, infection control, and continuity of care between inpatient and outpatient settings [19–21]. Problems associated hemodialysis session instability, as marked by the occurrence of intradialytic complications, have received less attention. Developing a patient-centered definition of hemodialysis session instability that takes into account patients’ perspectives and experiences is desirable in order to improve patient care [22]. Additionally, while key stakeholder organizations have called for greater patient involvement in safety [23–26], such efforts rarely consider patients’ perspectives regarding intradialytic complications [27]. Given these gaps, we used qualitative methods that permitted patients to express their experiences and perspectives in their own words to investigate patients’ definitions of an unstable hemodialysis session, or a “bad run” — a term that we found in previous fieldwork, validated by our patient study partners, that patients often use to describe hemodialysis sessions accompanied by complications. Leveraging the strength of qualitative methods for exploring new concepts, the study aimed to develop the patient-generated idea of a “bad run,” which can inform the selection of patient-centered outcomes for patient safety research. We also investigate patients’ beliefs regarding the causes of and solutions to bad runs so as to inform the design of patient safety interventions.

## Materials and methods

### Study design and conduct

This exploratory, cross-sectional study was conducted within a qualitative paradigm, with experiential and realist ontological orientations such that language was seen to capture “participants’ experiences of reality” [28]. With an inductive, thematic approach to analysis, the study was not initiated with a prior theoretical framework. Seventeen patients attended three focus groups from February to September 2017. The focus group method was chosen to foster experience-sharing between patients, and to facilitate comparisons between themselves, with the goal of eliciting a range of perspectives and interpretations. To supplement these data, eight patient advocate participants also completed an online survey populated with structured demographic questions, and open-ended questions regarding their perspectives on “bad runs.”

Study inclusion criteria included being an adult (aged 18 or older) hemodialysis patient, and/or an adult with hemodialysis experience who was also a peer mentor or advocate for other patients. Exclusion criteria included being under 18 years of age and not having hemodialysis experience. A Quota sampling [29] technique ensured variability in hemodialysis experiences based on residence in different parts of the United States. Selection of extreme cases [29] was used to identify eight additional patient advocates, who are individuals who speak out “on behalf of persons with chronic kidney disease (CKD) or on behalf of a CKD-related cause [30];” this resulted in a total of 25 patient participants. Advocates were recruited as they were expected to be sensitized to issues concerning patient well-being and thus provide rich data concerning the phenomenon of interest; these participants also served on Advisory or Steering Committees for a larger PCORI-funded cluster-randomized trial focused on session instability reduction (“Dialysafe [31]”) for which the authors are investigators and/or staff.

Recruitment of focus group participants took place via the National Kidney Foundation (NKF), dialysis facilities and patient advocacy organizations via three email lists (2336 subscribers), social media postings, telephone calls to three organizations that were asked to share study information with patients (patient nonprofit organization, end-stage renal disease (ESRD) care quality improvement organization, renal social workers association) and an email to current NKF peer mentors. Interested participants were asked to contact an NKF staff member (M.A.) to indicate their intention to attend. Given broader Dialysafe study goals in relation to peer mentoring intervention design to prevent hemodialysis complications, nine participants had experience as peer mentors as part of NKF’s patient peer mentoring programs and were recruited through this existing contact. For the surveys, all patients serving on the Dialysafe study Steering or Advisory Committees were invited to attend by email; all participants attempted to complete the survey, although one submission was incomplete. This resulted in eight usable surveys.

### Data collection

The principal investigator (PI, T.V.), a female PhD-trained professor of information science and public health, designed the focus group guides and surveys, and oversaw the complete process of data collection and analysis. A semi-structured focus group discussion guide was developed to address study research questions. A draft of the guide was shared with a steering committee (including two patient advocates, project staff and investigators) and a 20-person national study advisory committee (including seven patient advocates, and 13 clinicians and researchers), and then revised based on their feedback. The discussion guide elicited patients’ perspectives regarding what constitutes “bad runs”; their own experiences with bad runs in general and IDH in particular (important given its long-term effects); other patients’ experiences of bad runs that they may have witnessed; and perspectives on what works and does not work to prevent or ameliorate bad runs. Other issues probed but not discussed in this paper include why patients may or may not get involved in their care, and preferences for the design of a peer mentoring-based patient activation intervention focused on preventing hemodialysis complications. The same investigator (T.V.) and a female project manager with an MA in communication conducted the groups in person or via video conference, which lasted an average of 90 min. One patient (i.e., patients without peer mentor experience) and one peer mentor focus group (i.e., patients with peer mentor experience) were held face-to-face in a Northeastern and a Midwestern state, respectively. One videoconference-based focus group was also conducted to reach peer mentors from across the country. In each focus group, the facilitator posed questions to the group based on the main questions in the discussion guide, while following up with probes drawn from the discussion guide as necessary. Follow-up questions were posed to clarify points if needed, and the facilitator asked individual participants for their responses to questions if they had not yet spoken.

Neither facilitator had prior relationships with the focus group participants; they were introduced as researchers from the University of Michigan who were working with the National Kidney Foundation. The purpose of the focus groups was described as asking patients to share their experiences with dialysis treatments, with the goal of learning “…more about what makes for a ‘bad run’ on dialysis and how these bad runs could be avoided.” All in-person focus groups were conducted at an NKF office, while one was conducted on a HIPAA-compliant videoconference platform, Blue Jeans. In addition to the facilitator and focus group participants, a female social worker employed by NKF (M.A.) was present at all focus groups to assist with focus group logistics (e.g., tracking attendees, administering $20 honoraria, arranging travel for the face-to-face groups). She had prior relationships with the participants who served as peer mentors in the NKF program.

Subsequently, to enrich the findings and help achieve data saturation, eight patient advocate participants completed an online survey using the Qualtrics survey platform [32]. Through their roles on the Dialysafe study’s national Advisory or Steering Committees, these “extreme case” [29] advocates had previously participated in a Delphi panel to inform the design of the overall Dialysafe study. This panel, which also included clinicians and researchers, involved evaluation of the scientific evidence concerning the prevalence and correlates of complications during dialysis, as well as candidate intervention strategies. Patient advocates’ roles in the panel were to evaluate the relevance of the evidence in light of patient experiences. While patients discussed their personal experiences of bad runs during the Delphi panel, it had not been designed to systematically capture these patient advocates’ definitions of “bad runs” and their experiences with them. Therefore, a follow-up survey was designed to more thoroughly gather each of these patient advocates’ perspectives in their own words. Participants were invited to participate by email, completed a demographic survey, and completed open-ended questions drawn from the focus group discussion guide, including what they think makes for “bad run” on dialysis, and personal experiences with bad runs including what they did in response and how others may have helped them. After the surveys, data saturation had been reached; that is, new data were repeating the experiences and perspectives found previously-gathered data [33].

### Data analysis

Focus group audio recordings were transcribed verbatim and verified by the study project manager. In response to each of the research questions (definitions of “bad runs”, perceived causes, and solutions), all data from the focus groups and open-ended survey questions were analyzed inductively by two investigators, the PI and a postdoctoral fellow (P.K.), through descriptive coding [34] and in vivo coding [35] (i.e.., identified patient language regarding “bad runs”) using Excel. These inductive codes were based on what participants said (i.e., they were semantic codes [28]) and added to a codebook to ensure consistency in first-round coding. Next, thematic analysis was performed to examine the topic(s) and meaning of coded data to capture patterns [36]; this involved reviewing existing codes, dividing/collapsing and clustering them around central ideas regarding patient bad run definitions with the aid of Excel tables. At this stage, codes focused on perceived solutions were clustered around the concepts of “patient-driven” and “clinician-driven” solutions, and various symptoms were combined under the theme of the type of experience that was a “bad run.” For example, codes regarding “extreme fatigue,” “sweating,” and “lightheadedness and blacking out” were clustered under “Crashing,” which was initially an in vivo code based on patient language for the experience of low blood pressure. Analytic memos were written to further develop emergent themes. Four themes emerged from these analyses, representing the achievement of inductive thematic saturation such that no new codes and themes were being identified in the data at the end of the second round of analysis [33]. Member checking was completed when a draft of the paper was given to two patient participants who were members of the Dialysafe project Steering Committee for review. These patients confirmed the salience of the themes, and related interpretations.

## Results

### Characteristics of participants

Most participants were between 45 and 64 years of age (52%) (*M* = 55.56, age range: 27–71 years), and the majority White (44%) or African American (44%) (Table 1). More than half of our patients had completed a Bachelor’s degree or higher level of education (56%). Patients came from five regions of the US, with the majority from the Northeast (40%) and the Midwest (32%). Participants had received dialysis for an average of 10.6 years.

**Table 1:** Participant Demographics

Demographics (*N* = 25)	
Age group	
≤ 45 y	5 (20)
46–64 y	13 (52)
≥ 65 y	7 (28)
Gender	
Male	11 (44)
Female	14 (56)
Race/Ethnicity	
White	11 (44)
Black or African American	11 (44)
American Indian or Alaska Native	1 (4)
Asian	1 (4)
Native Hawaiian or Pacific Islander	0
Other	1 (4)
Educational Level	
High school graduate or equivalent (GED)	1 (4)
Some college	5 (20)
College degree (e.g., AA, AS, BA, BS, MA, MS, PhD, MD, JD)	19 (76)
Current Employment	
Working full-time (30 h or more)	5 (20)
Working part-time (less than 30 h)	3 (12)
Unemployed	4 (16)
Retired	6 (24)
Other	4 (16)
Geographical Region	
Northeast	10 (40)
Northwest	1 (4)
Midwest	8 (32)
Southwest	3 (12)
Southeast	3 (12)
Monthly Income	
$0–$1000 ($0–$12,000 per year)	3 (12)
$1001–$2000 ($12,012–$24,000 per year)	8 (32)
$2001–$3000 ($24,012–$36,000 per year)	3 (12)
$3001–$4000 ($36,012–$48,000 per year)	4 (16)
$4001–$5000 ($48,012–$60,000 per year)	1 (4)
$5001 + (over $60,000 per year)	3 (12)
Years Receiving Dialysis (as of 2017)	
Mean (SD); Range	10.63 (SD = 7.37); 23
Years Since Finding Out Kidney Disease (as of 2017)	
Mean (SD); Range	20.1 (SD = 14.06); 48
Has had a kidney transplant	7 (28)
Kidney transplant is still functioning	6 (24)

*Note:* Percentages are in parentheses

### Definitions of a “bad run”

From patients’ perspectives, “bad runs” involved unusually severe discomfort or unanticipated events that interfered with receipt of hemodialysis therapy. These included four major negative experiences identified by patients: 1) cramping; 2) “crashing”; 3) cannulating-related problems; and 4) clotting of the dialysis circuit or vascular access. Below, we summarize how patients described these experiences, and what they perceived as causes of and solutions to them.

#### Cramping

As Table 2 shows, patients describe cramping as severe pain in various parts of their bodies. Limbs such as legs and arms were the most common locations in which cramping occurred, followed by abdomens, necks, and feet. According to patients’ descriptions, cramping can be so severe that it feels like “cardiac arrest”, or may lead to passing out from pain. The duration of cramps also contributed to bad runs. Patients described lasting pain in their muscles and a lack of relief from that pain. Patients mentioned that cramping developed during dialysis could last from several hours to several days after their session was completed.

**Table 2:** Patient quotes regarding cramping

Cramping	
**Experience**	
*Severe Pain in various parts of body* “A type of throbbing pain or cramp that makes a bad run for me.” “My life is gonna be over. And I tell ya they are in my arms, in my legs. They’re in my stomach.” “Cramping like it will break bones...pain enough to consider leaving early or not return”	
*Lasting Pain* “I used to cramp and I would go home…it’s a Friday, until that Sunday night sometimes or early that Monday, I wouldn’t feel relief.”	
**Perceived Causes**	
*Incorrect post-dialysis target weight* “…the cramping came from my gaining weight and needing my dry weight to be increased and they were taking too much off.”	
*Fluid removal volume and speed* “…a bad run would be…cramping being on the machine, taking off too much fluid, too fast.”	
*Patients consuming too much fluid* “…I …caused a lot of problems for myself with bad runs by coming in after having drank too much in between treatments and would sit there and cramp severely.”	
*Low blood pressure* “Low pressure oftentimes leads to cramping. Buildup of lactic acid…hurts like hell.”	
**Perceived Solutions**	
*Patient-driven*	
*Involvement in decisions about fluid removal* “Control fluids to avoid cramping, self-managing how much fluid is taken off in a dialogue with the tech…”	
*Tracking weights* “Get a little notebook, write it down. What you weigh when you come in and what you weight when you go out. And then you start figuring it out. And you’ll have a better idea of how much fluid you’re ingesting…”	
*Dietary Intervention* “…I could walk out like I’m a brand new person because that salt works. It does wonders…no chips.”	
*Clinician-driven*	
*Fluid administration, dialysis session interruption* “Received extra saline, sometimes turned off the machine, or came off the machine.” “they can’t give you so many fluids to stop it because it defeats the purpose of why you came to dialysis.”	
*Manual Intervention* “…the nurses and technicians try. They try to massage they try to do what they can. They’re hurting from seeing people hurting… it’s very immediate. So they try what they can. But they can’t bring it down so quickly.”	

##### Perceived causes

Patients attributed cramping partly to clinicians’ decisions (Table 2). Such decisions included not changing their post-dialysis target weight (“dry weight”) often enough and removing too much fluid too quickly. Patients also believed that cramping was linked to their own self-care behavior; specifically, patients reported that cramping could follow from drinking too much, or not drinking enough, fluid between sessions.

##### Perceived solutions

To prevent cramping, patients advocated several patient-driven solutions. They emphasized the importance of having conversations with clinicians regarding their fluid removal goals. They also discussed the value of tracking and recording their own weights before and after each session, and notifying staff if their body weight had changed in a way that might influence their post-dialysis target weight.

To ease the pain from cramping, patients have adopted their own post-dialysis dietary interventions, including eating salt or salty snacks such as potato chips, though there were differing perceptions as to whether these approaches actually work, and how much salt or which type of salty food was most effective.

According to patients, clinician-directed efforts to ease cramping commonly included giving them intravenous fluids. However, some were concerned about the consequences of this strategy. Patients also reported interrupting the dialysis session to ease cramping. Additionally, manual interventions such as pressing on feet or massage could be helpful, but it took longer to see an effect than with other approaches.

#### Crashing

Crashing was typically experienced as sudden and rapid in progression. As shown in Table 3, patients reported that common symptoms of low blood pressure include extreme fatigue, nausea and vomiting, lightheadedness, “blacking out,” and sweating. More severely, symptoms occasionally included uncontrollable vomiting, and temporarily losing the ability to see, walk, hear, or speak normally.

**Table 3:** Patient quotes regarding crashing

Crashing	
**Experience**	
*Extreme fatigue* “…really completely exhausted. Not the type of exhausted that you feel like you could really just lay down and recover from…you just feel like you’re done for the rest of the day.”	
*Nausea and Vomiting* “Cause the pressure - when it drops and you feel that weight, you feel nauseous too at the same time…. You start throwing up immediately. I mean uncontrollably sometimes.”	
*Lightheadedness and Blacking out* “But that passing out thing, you really can’t see or hear. You think you’re responding but you’re not. And people say, “you ain’t saying nothing”. “And just the feeling of, like getting close to losing consciousness or completely losing consciousness is just scary.” “It feels like the world is closing in and my head got extremely light.”	
*Sweating* “...skin became sweaty and clammy.”	
*Lasting Symptoms* “…it first started dropping, it started in the dialysis center. When I first got on and everything it would always drop there. Then after a while, it kept dropping even when I wasn’t there.”	
**Perceived Causes**	
*Incorrect post-dialysis target weight* “So they did what they were supposed to do - take the fluids off and bring me to my dry weight. But my dry weight was too low, I had gained weight.”	
*Fluid gains* “It may be from gaining too much fluid.”	
*Treatment time* “…there are times when I have to run normal dialysis during the day, which is the four-hour dialysis. I find that I have more low blood pressure episodes on that type of dialysis than nocturnal.”	
**Perceived Solutions**	
*Patient-driven*	
*Fluid and dietary adherence* “...monitor their diet…make sure that they’re not going over their daily recommended salt intake.” “…gain less weight before they go in for each session.”	
*Timing of eating* “...if you haven’t had a decent enough base of carbohydrates, to see you through dialysis, that’s going to lead to crashing.”	
*Notifying staff of symptoms to facilitate early intervention* “…watch for signs of hypotension and telling the tech to stop taking fluid off.”	
*Change Dialysis Modality* “I would recommend…switching to nocturnal hemodialysis, because I have less low blood pressure episodes on that.”	
*Dietary Intervention* “I had a nurse that…suggested drinking a cup of cold ice water, and that actually helps me recover a lot quicker from the low blood pressure episodes.”	
*Clinician-driven*	
*Change Timing of Blood Pressure Medication* “My doctor and I played around with my blood pressure medication, so basically on dialysis nights, I just hold my blood pressure medication….”	
*Change Fluid Removal Goal* “…they’ll take less of, they’ll do anything they can, to keep you from crashing.”	
*Slowing or stopping ultrafiltration* “…cut back the goal on the machine or just stopped the machine from pulling more fluid depending on the severity of the issue.”	
*Fluid Administration* “…resolving the low blood pressure problem..[i]t would either be fluid or it would be the broth.”	
*Trendelenburg position* “Your blood pressure could drop…they have to tilt your chair back and try and give you fluid.”	

The majority of crashing experiences began and ended within the dialysis session; however, it could also occur during the session followed by symptoms that continued afterwards. This caused short-term complications such as having trouble staying awake while driving home, and a long recovery time after dialysis.

##### Perceived causes

Patients believed that crashing was caused by poor fluid management on the part of clinicians or patients. This could be related to post-dialysis target weight miscalculation by clinicians or patients consuming too much fluid between sessions. One patient blamed IDH on hemodialysis in general, instead advocating home hemodialysis with longer treatment times as an alternative.

##### Perceived solutions

Patients believed there were things they could do in the interdialytic period to prevent crashing. They felt that good control of diet, salt, and fluid were key factors. This included closely following the daily recommendations for salt intake, and limiting weight gain. As patients noted, these actions would decrease the likelihood of edema and fluid retention. In the intradialytic period, patients indicated it is important to: (1) avoid eating while dialyzing, (2) identify early symptoms of low blood pressure (such as increased body temperature and feelings of nausea), and notify staff of them, and (3) change their dialysis modality. Additionally, patients mentioned having cold ice water and salty snacks to help speed up post-crash recovery.

To prevent crashing, two patients mentioned that clinicians have asked them to skip or delay their blood pressure medications on dialysis days. Other patients said that their clinicians lowered their fluid removal goal in a given session. To prevent or ease crashing, patients also reported clinicians have: slowed or stopped ultrafiltration, given them fluid intravenously or orally, and/or tilted their chair to place them in the Trendelenburg position. Patients complained about the first two practices due to the receipt of insufficient treatment and the risk of fluid overload.

#### Cannulating-related problems

Patients define “bad sticks” as access area pain; such pain could last for the entire dialysis session (Table 4). A related problem was that difficult cannulation could take a long time, resulting in shortened treatment time. Infiltration was also part of a cannulation-focused bad run.

**Table 4:** Patient quotes regarding cannulating-related problems and clotting of the dialysis circuit or vascular access

**Cannulating**	
**Experience**	
*Pain* “Have pain in the access area.” “…if they hit a nerve…it causes pain throughout the entire run, which is really not comfortable.”	
*Prolonged Cannulation* “Do we actually lose time? If it takes a half hour to stick us, do we cut that time off”	
*Infiltration* “Bad sticks that lead to infiltration, short treatment or almost no treatment.” “I had a bad run when I was stuck wrong and my blood was oozing out of my venous line and it came out even more till we had to stop the treatment….”	
**Perceived Causes**	
*Staff skill or technique* “One time I had the needles put too close together and after all…three and a half hours of treatment, I was never dialyzed…that was a bad thing.”	
*Staff knowledge of patient* “…you have some techs that are really familiar with you, they can…get you in, Some techs you’re just kinda nervous about ‘cause they may or may not infiltrate you, which means you can’t dialyze that day.”	
**Perceived Solutions**	
*Patient-driven*	
*Requesting a different staff member* “…patients ask other techs to see if they can put you on as opposed to that particular tech that you’re assigned to.”	
*Change access type* “It could also involve being infiltrated by the person cannulating me, which is part of the reason I switched to buttonholes.”	
*Clinician-driven* “Ice pack on the infiltration…”	
**Clotting**	
**Experience**	
*Clotting of Access* “Sometimes it could be a problem with your access site, it might clot off for whatever reason.” “…I go in early, go in and they take the stethoscope, listen for the thrill sound if it’s running and if it’s not that’s a bad run; cause I can’t run. I now have to go in and get a de-clot.”	
*Frequency* “Yeah. Two weeks in a row.” “It could also involve clotting, which rarely occurred.”	
*Shortened or Missing Treatment* “Sometimes it could be a problem with your access site, it might clot off for whatever reason and most of the time people who end the treatment early just don’t feel well, they just wanna get off the machine.”	
**Perceived Causes**	
*Blood Disorder* “The problem is, is that my graft doesn’t follow the rules. I follow them, my graf doesn’t. …because I have a blood disorder, that clots.”	
**Perceived Solutions**	
*Patient-driven* None	
*Clinician-driven*	
*Surgery* “I have to wait till the surgeon can get me in, to do a de-clot.”	
*Medication* “I’m not sure if all of the patients are having to go without heparin if they’re on Coumadin now, but I believe that’s what our unit was following. They have to give me heparin.”	

##### Perceived causes

The most common patient explanation for bad sticks concerned dialysis staff skill or technique. Patients indicated poor cannulation technique, such as needles placed too close together, could be a cause. Additionally, patients believed that they had better experiences with cannulation when a dialysis team member knew them well; one patient said that the high staff turnover rate at his dialysis facility impeded the development of such familiarity. One patient also saw bad sticks as originating from their body’s unique characteristics.

##### Perceived solutions

Patients advocated switching to a provider with whom one feels comfortable, or changing the access cannulation to the buttonhole technique to prevent infiltration. One patient also learned to self-cannulate. One patient brought up using an ice pack as a clinician-driven approach to addressing bad sticks.

#### Clotting of Dialysis circuit or vascular access

The frequency of clotting varied among individuals. Among the three patients who reported a clotting experience, two identified clotting of the access site as an event that made for a bad run, though one patient mentioned clotting of the dialysis line (Table 4). For patients, one difficulty with clotting was that it could result in missing or having shortened sessions. Loss of blood during line clotting was a concern for one patient.

##### Perceived causes

Patients in our study did not point to specific causes of clotting other than idiosyncratic factors related to their own bodies such as a difficult access or a blood disorder.

##### Perceived solutions

No patient-directed efforts were mentioned to prevent clotting. One patient described having de-clotting surgery and/or getting the blood thinner to address clotting.

## Discussion

Patients define bad runs as dialysis sessions in which they experience severe discomfort or unanticipated events that interfere with their ability to receive hemodialysis. Bad runs were characterized by cramping, low blood pressure (“crashing”), cannulation-related difficulties (“bad sticks”), and clotting of the dialysis circuit or vascular access. Notably, infiltration of the access site, clotting of the dialysis circuit, IDH and cramping are relatively common [5, 37, 38]. Patients highlight the suffering they experience during bad runs, with some issues such as cramping and fatigue often persisting beyond the dialysis session itself. This aligns with prior research emphasizing patients’ priorities for finding solutions to cramping and fatigue [39]. Patients perceived both cramping and crashing as experiences to be explained by both patient and clinician behavior, with patient fluid consumption and providers’ aggressive fluid removal and/or inappropriate post-dialysis target weight being key perceived culprits. In contrast to cramping and crashing, patients perceived that the responsibility for cannulation-related problems lay entirely with dialysis facility staff; these problems include painful needle insertion and misplacement of needles. Consequently, patients used strategies such as asking for different staff or self-cannulation as solutions. Patients expressed concern about “bad runs” on their ability to receive enough dialysis and achieve fluid balance, since shortened sessions reduced ultrafiltration and administration of additional fluid was often the result.

National and international stakeholder organizations have advocated for greater patient involvement in safety [23–26]. However, previous initiatives have met with mixed success, and few patient safety interventions have been shown to improve safety in outpatient settings [40]. We posit that one of the reasons for this can be traced to the fact that, aside from error reporting systems [25, 26], few initiatives promoting patient safety begin directly from patients’ concerns and experiences — thus providing limited incentives for patients to become involved in safety. Results of this study suggest a need for patient safety efforts to focus more on common complications in dialysis care that comprise patients’ definitions of session instability or “bad runs”, thus extending beyond more rare events such as patient falls or staff errors [37, 41]. Results of this study also show that, in an outpatient hemodialysis context, it could be viable for patients to be more involved in their safety, with a focus on prevention and management of dialysis session instability. Findings suggest that patients are concerned about dialysis session instability and the suffering that they experience from such instability. Moreover, they believe that much of the suffering they experience during bad runs is avoidable, and were often taking actions to prevent bad runs on their own, and in collaboration with their dialysis team members. Accordingly, future patient safety interventions should be specifically designed to reduce the occurrence of “bad runs,” and these interventions should be evaluated against patient-centered outcomes that are operationalized as the occurrence of these untoward experiences during hemodialysis. Such patient-centered outcomes for the patient safety domain can complement efforts such as the standardized outcomes in nephrology-hemodialysis (SONG-HD) [42], which has a broader focus than the specific problem of patient experiences of dialysis therapy.

Beginning from patients’ perspectives also provides insights into ways in which patients can be involved in improving the stability of their hemodialysis sessions. First, some patients talked about bad runs originating from difficulties related to their post-dialysis target weight and fluid gain between sessions. To address this, patients commonly described efforts to adhere to sodium and fluid restrictions. Additionally, several individuals talked about monitoring their weight and actively participating in clinical decisions regarding their fluid removal goals. Second, three patients in our study believed it is critical to recognize early symptoms of crashing, and to react by notifying their dialysis care providers. Third, our data show that patients can be involved in easing cramping and crashing, as some already consume salt or salty snacks to address these problems; scientifically-validated solutions that would help without later increases in thirst would also be desirable. Fourth, patients already actively try to improve their cannulation experiences by communicating with staff about their needs and building relationships with them. Another perhaps under-utilized method, even in our activated sample, is self-cannulation. With regard to clotting, potential patient roles remain unclear, as patients did not see a role for themselves in its prevention.

To facilitate patient involvement in dialysis session stability, there is a need to focus on patient education and counseling on topics related to their concerns, which could potentially benefit all non-cognitively impaired patients regardless of their socio-economic status. Programs addressing sodium and fluid restrictions, as well as self-management through weight and fluid removal tracking, could prepare patients to notify their dialysis care providers if they have gained or lost weight so that their post-dialysis target weight can be revisited in a timely fashion. Support for home blood pressure monitoring, which has been successful in several contexts [43, 44], could also help patients to identify irregularities in blood pressure of which their providers should be aware. Education and support in methods of collaboration and assertive communication [45, 46] could also assist patients with being more involved in decisions about their care, including fluid removal. Furthermore, patients could be trained to identify early symptoms of cramping or crashing, and to notify their providers to facilitate early intervention. Such training may be especially important given that patients may not always report negative experiences such as symptoms to providers [47]. Communicating with staff regarding cannulation, and self-cannulation are also areas of potential focus. Additionally, we note that patients’ accounts of bad runs were laden with descriptions of physical pain. Patients wanted to know what else they could do to ease pain from cramping as they expressed concerns regarding common methods for handling their pain. Given the lack of clinical guidelines for chronic pain management among dialysis patients [48], patient education and counseling could also focus on techniques such as meditation [49, 50] and intradialytic exercise [51].

The study has two main limitations. First, patient participants, including patient advocates, had relatively higher levels of education and more years of dialysis experience than most US dialysis patients. Accordingly, they were likely more activated and involved in their care than average, and perhaps more sensitized to dialysis session instability than the larger dialysis patient population. Some patients may ignore bad runs when they occur (for example, intradialytic hypotension while they are asleep) or be less knowledgeable about causes and solutions for the issues arising during “bad runs.” Others may have become accustomed to “bad runs” and expect that this is how they are to feel during or after hemodialysis treatments. Second, bad run experiences shared by participants likely emphasized extreme instances — an example of recall bias. Therefore, less extreme experiences of IDH were possibly not captured — although arguably these may not have been identified as “bad runs.” Nevertheless, the perspectives offered in this study provided a rich view of unstable dialysis sessions, and of potential strategies for facilitating patient involvement in preventing complications.

## Conclusions

To advance patient safety research in the hemodialysis context, we examined patients’ definitions of an unstable hemodialysis session (or “bad run”) through focus groups and surveys. Their accounts focused on four major, negative experiences: 1) cramping; 2) crashing; 3) cannulating-related problems; and 4) clotting of the dialysis circuit or vascular access. We also outlined patients’ perceptions of the causes of, and potential solutions for, these phenomena. Our study findings point to several areas in which patients can be supported in becoming more involved with improving the stability of their hemodialysis sessions. Patient perspectives also identified areas upon which dialysis care providers should focus to improve patient experiences on dialysis.

## Supplementary information

**Additional file 1: Supplementary materials.** Cramping, Crashing, Cannulating, and Clotting: A Qualitative Study of Patients’ Definitions of a “Bad Run” on Hemodialysis: Study Instruments. Single file containing: (1) Patient Focus Group Discussion Guide; (2) Peer Mentor Focus Group Discussion Guide; (3) Survey for Patient and Peer Mentor Focus Group Participants; and (4) Patient Advocate Survey.

## Data Availability

The datasets generated and/or analysed during the current study are not publicly available due privacy concerns.

## Abbreviations

CKD: Chronic Kidney Disease
ESRD: End-stage renal disease
HRQOL: Health-Related Quality of Life
IDH: Intradialytic Hypotension
NKF: National Kidney Foundation
PI: Principal Investigator
SONG-HD: Standardized Outcomes in Nephrology-Hemodialysis project
