# Supplementary material for: Cramping, crashing, cannulating, and clotting: a qualitative study of patients’ definitions of a “bad run” on hemodialysis
Source: BMC Nephrol. 2020 Feb 27;21:67. doi: 10.1186/s12882-020-01726-8 (PMC7045425; doi:10.1186/s12882-020-01726-8)
Supplement: Supplementary file 1 — Additional file 1: Supplementary materials. Cramping, Crashing, Cannulating, and Clotting: A Qualitative Study of Patients’ Definitions of a “Bad Run” on Hemodialysis: Study Instruments. Single file containing: (1) Patient Focus Group Discussion Guide; (2) Peer Mentor Focus Group Discussion Guide; (3) Survey for Patient and Peer Mentor Focus Group Participants; and (4) Patient Advocate Survey. [file 12882_2020_1726_MOESM1_ESM.pdf]

Cramping, Crashing, Cannulating, and Clotting: A Qualitative Study of Patients' Definitions of a "Bad Run" on Hemodialysis

Study Instruments

Contents

1. Patient Focus Group Discussion Guide
2. Peer Mentor Focus Group Discussion Guide
3. Survey for Patient and Peer Mentor Focus Group Participants
4. Patient Advocate Survey

Dialysafe Patient Focus Groups: Session 1

[Note: in keeping with the principles of qualitative research, these questions may evolve through the course of this research. However, this interview guide shows the possible range of questions to be asked.]

1) In your opinion, what makes for "a bad run" at dialysis?

Probes

a) Have you ever had a "bad run" at dialysis? What happened?
b) Have you ever seen other people have "bad runs" at dialysis? What happened?
c) Did anyone else help you during or after? What did they do?

2) Has a member of your healthcare team ever told you that you had low blood pressure or hypotension during dialysis?

Probes

a) What did it feel like? What did you do? What happened afterwards?
b) How often does this happen to you?
c) Did anyone else help you during or after? What did they do?

3) Imagine that you are speaking with a patient who is relatively new to dialysis. They complain that they are having a lot of bad runs because of low blood pressure, and they want your advice about how to make them stop. What would you tell them are the best ways to make their dialysis treatments better?

Follow-up questions

For each approach mentioned, ask:

a) What makes you say that ____ would be a good idea?
b) Have you tried ___?
c) What happened ___?
d) Has someone else you know tried ___?

4) Imagine that you are still speaking with the patient who is relatively new to dialysis who is having a lot of bad runs because of low blood pressure. Now they ask you what DOESN'T work to stop bad runs. What would you tell them are things that WON'T work to make dialysis treatments better?

Follow-up questions

For each approach mentioned, ask:

a) What makes you say that ____ would not work?
b) Have you tried ___?
c) What happened ___?
d) Has someone else you know tried ___?

5) **People who are on dialysis are told to limit how much fluid they drink and eat. How easy or hard has limiting fluid been for you?**

Probes:

a) What makes it easy to do this?
    i. Are there particular times and places when it is easier to do this? If so, what are they?
b) What makes it hard to do this?
    ii. Are there particular times and places when it is harder to do this? If so, what are they?
c) Do you do anything to help you keep track of how much fluid you drink or eat? If so, what do you do?

6) **Imagine that you are speaking with another patient who is relatively new to dialysis. They say that a nurse at the clinic told them that they are gaining too much weight between treatments because of how much fluid they are drinking and eating. What would you tell them are the best ways to limit fluid intake?**

Follow-up questions

For each approach mentioned, as;

a) What makes you say that ____ would be a good idea?
b) Have you tried ___?
c) What happened ___?
d) Has someone else you know tried ___?

7) **People with kidney disease can get involved in their health care in a number of ways, including sharing their preferences when asked, or making requests about their care at their own initiative. For example, a patient might ask for a specific post-dialysis target weight or that a certain amount of fluid be removed from them in their dialysis treatment. Do patients at your facility, including you, share their preferences or make requests about their dialysis care now? If so, tell me about a time when this happened.**

Follow-up questions

a) What was the preference or request?
b) How did dialysis staff members react?
c) If you happen to know, was the patient's request granted?
d) Did a dialysis staff member do anything to encourage the patient to provide input?
e) Did a dialysis staff member do anything to let the patient know that they were open to patient input?

8) **Sometimes people on dialysis prefer NOT to get involved in their care. What are the reasons why a patient might not want to be involved?**

Follow-up questions

a) Are there specific situations in which you think patients might be more likely to prefer not to be involved?
b) Do dialysis care providers ever discourage patients from being involved in their care? If so, what have you seen or experienced of this?

9) Many dialysis patients have family or friends with whom they regularly interact. Thinking about those people in your life, is there anything that you wish they knew about your experience with dialysis?

Probes:
a) Why do you wish they knew that?
b) Have you ever shared this with your family or friends? What happened?
    - If you haven't shared it with your family or friends, what keeps you from sharing it with them?

10) Still thinking about your family or friends, is there anything that you wish they would do to help you to deal with dialysis?

Probes:
a) Is there anything they could do to help you after a bad run?
b) Is there anything they could do to help you keep from having a bad run?
c) Is there anything they could do to help you limit how much fluid you drink or eat?
d) Is there anything they could do to help you eat less salt?
e) Is there anything they could do to help you interact with your hemodialysis providers?

11) Do you remember anything that another person with kidney disease has said or written that was very helpful to you, or that made a significant impression on you?

Probes:
a) If so, what was it?
b) How did it help you?

12) We are developing a program for patients to help them have fewer dialysis sessions with low blood pressure, or more "good runs". The program will involve matching patients with other patients who have trained to be peer mentors, and having the patient and peer mentor pair talk about six times. In your opinion, what kinds of issues should those peer mentoring discussions deal with, and what specifically would you like the peer mentor to say or do in them?

13) To go along with the peer mentoring sessions, we are thinking about giving people information about low blood pressure and fluid management that they can look at during their dialysis sessions. This information would be in an application on a tablet computer, and would include videos, quizzes and other interactive content. How much do you think people on dialysis would use that application?

Probes:

a) Why do you think they would look at the application that much?
b) What do you think might be attractive about an application like that?
c) What do you think might make them not want to us an application like that?
d) What could be done to make it more appealing?

Participatory Design Exercises

Collage/Drawing or low-tech prototype

- Provide participants with glue, scissors, markers, pens, paper
- Ask participants to draw a concept, then explain to group.

Instructions:

Part 1: Imagine that you have a peer mentor and that they live far away from you, so you are going to communicate them using a tablet computer. Please draw what you want to see on the screen when communicating with your mentor, and what they see when they are communicating with you.

Part 2: Imagine you are going use an application on a tablet computer to learn about how to have more “good runs” on dialysis. Please draw what you want the screen to look like and what would you want it to include.

Dialysafe Peer Mentor Focus Groups: Session 1

[Note: in keeping with the principles of qualitative research, these questions may evolve through the course of this research. However, this interview guide shows the possible range of questions to be asked.]

1) In your opinion, what makes for "a bad run" at dialysis?

<u>Probes</u>

a) Have you ever had a "bad run" at dialysis? What happened?
b) Have you ever seen other people have "bad runs" at dialysis? What happened?
c) Did anyone else help you during or after? What did they do?

2) Has a member of your healthcare team ever told you that you had low blood pressure or hypotension during dialysis?

<u>Probes</u>

a) What did it feel like? What did you do? What happened afterwards?
b) How often does this happen to you?
c) Did anyone else help you during or after? What did they do?

3) Imagine that you are speaking with a patient who is relatively new to dialysis. They complain that they are having a lot of bad runs because of low blood pressure, and they want your advice about how to make them stop. What would you tell them are the best ways to make their dialysis treatments better?

<u>Follow-up questions</u>
For each approach mentioned, ask:
a) What makes you say that ____ would be a good idea?
b) Have you tried ___?
c) What happened ___?
d) Has someone else you know tried ___?

4) Imagine that you are still speaking with the patient who is relatively new to dialysis who is having a lot of bad runs because of low blood pressure. Now they ask you what DOESN'T work to stop bad runs. What would you tell them are things that WON'T work to make dialysis treatments better?

<u>Follow-up questions</u>
For each approach mentioned, ask:
e) What makes you say that ____ would not work?
f) Have you tried ___?
g) What happened ___?
h) Has someone else you know tried ___?

5) **Imagine that you are speaking with another patient who is relatively new to dialysis. They say that a nurse at the clinic told them that they are gaining too much weight between treatments because of how much fluid they are drinking and eating. What would you tell them are the best ways to limit fluid intake?**

<u>Follow-up questions</u>

For each approach mentioned, as;

a) What makes you say that ____ would be a good idea?
b) Have you tried ___?
c) What happened ___?
a) Has someone else you know tried ___?

6) **People with kidney disease can get involved in their health care in a number of ways, including sharing their preferences when asked, or making requests about their care at their own initiative. For example, a patient might ask for a specific post-dialysis target weight or that a certain amount of fluid be removed from them in their dialysis treatment. Have you ever shared your preferences or made requests about your dialysis care? If so, tell me about a time when this happened.**

<u>Follow-up questions</u>

a) What was the preference or request?
b) How did dialysis staff members react?
c) Was your request granted?
d) Did a dialysis staff member do anything to encourage the patient to provide input?
e) Did a dialysis staff member do anything to let the patient know that he or she was open to patient input?

7) **Sometimes people on dialysis prefer NOT to get involved in their care. What are the reasons why a patient might not want to be involved?**

<u>Follow-up questions</u>

a) Are there specific situations in which you think patients might be more likely to prefer not to be involved?
b) Do dialysis care providers ever discourage patients from being involved in their care? If so, what have you seen or experienced of this?

8) **What made you want to be a peer mentor?**

9) **What part of the peer mentor experience, if anything, do you find to be the most rewarding?**

10) **We are developing a program for patients to help them have fewer dialysis sessions with low blood pressure, or more "good runs". The program will involve matching patients with other patients who have trained to be peer mentors, and having the patient and peer mentor pair talk about six times. In your opinion, what kinds of**

issues should those peer mentoring discussions deal with, and what specifically would you like the peer mentor to say or do in them?

11) **Still thinking about the peer mentoring sessions that try to help patients have fewer dialysis sessions with low blood pressure, what do you think you would need as a peer mentor to feel confident discussing issues related to low blood pressure?**

    Probes
    a) What kind of training would be needed?
    b) What kind of support would be needed?

12) **To go along with the peer mentoring sessions, we are thinking about giving people information about low blood pressure and fluid management that they can look at during their dialysis sessions. This information would be in an application on a tablet computer, and would include videos, quizzes and other interactive content. How much do you think people on dialysis would use that application?**

Probes:
a) Why do you think they would look at the application that much?
b) What do you think might be attractive about an application like that?
c) What do you think might make them not want to us an application like that?
d) What could be done to make it more appealing?

13) **Participatory Design Exercises**

Collage/Drawing or low-tech prototype
- Provide participants with glue, scissors, markers, pens, paper
- Ask participants to draw a concept, then explain to group.

Instructions:

Part 1: Imagine that you are a peer mentor and your mentee lives far away from you, so you are going to communicate with them using a tablet computer. Please draw what you want to see on the screen when communicating with your mentor, and what they see when they are communicating with you.

Part 2: Imagine your mentee has an application on a tablet computer to help them learn about how to have more "good runs" on dialysis. This application is meant to be used to reinforce what you talk about in your peer mentoring sessions. Please draw what you want the screen to look like and what would you want it to include.

Part 3: Imagine that you have access to an online community with other peer mentors who are helping patients learn how to have more "good runs" on dialysis. This online community is meant to help peer mentors support one another and keep learning to help them to a good job with peer mentoring. Please draw what the online community would look like, and how it would work.

ID #: ___________

Demographic Survey

Focus group participants: Patients and Peer Mentors

These questions give us some background information about you.

1. **What is your birth month? __________ What is your birth year? __________**

2. **What is your gender?**
   - ❑ Male
   - ❑ Female
   - ❑ Other_______________________

3. **What is your race? (Check all that apply)**
   - ❑ White
   - ❑ Black or African American
   - ❑ American Indian or Alaskan Native
   - ❑ Asian
   - ❑ Native Hawaiian or Pacific Islander
   - ❑ Other__________________________

4. **If you selected "Asian," with which Asian ethnicity do you identify? (Check all that apply)**
   - ❑ Asian Indian
   - ❑ Chinese
   - ❑ Filipino
   - ❑ Japanese
   - ❑ Vietnamese
   - ❑ Korean
   - ❑ Other__________________________
   - ❑ Not applicable

5. **If you selected "Native Hawaiian or Pacific Islander," with which ethnicity do you identify? (Check all that apply)**
   - ❑ Native Hawaiian
   - ❑ Guamanian or Chamorro
   - ❑ Samoan
   - ❑ Other__________________________
   - ❑ Not applicable

6. **Are you Hispanic, Latino/a, or Spanish Origin (Check all that apply)?**
   - ❑ No, not of Hispanic, Latino/a, or Spanish origin
   - ❑ Yes, Mexican, Mexican American, Chicano/a
   - ❑ Yes, Puerto Rican
   - ❑ Yes, Cuban
   - ❑ Yes, another Hispanic, Latino/a, or Spanish origin

7. **What is your postal zip code? ___________________**

8. **What is the highest degree or level of school that you have completed? (check one)**
   - ❑ Grade 8 or less
   - ❑ Grades 9 to 12, no diploma
   - ❑ High school graduate or equivalent (GED)
   - ❑ Some college
   - ❑ Associate degree (e.g., AA, AS)

- ❑ Bachelor's degree (e.g., BA, BS)
- ❑ Graduate degree (e.g., MA, MS)
- ❑ Professional degree (e.g., MD, JD)

9. What is your current employment activity? (check all that apply)
- ❑ Working full-time (30 or more hours)
- ❑ Working part-time (less than 30 hours)
- ❑ Full-time student (12 or more credits)
- ❑ Part-time student (less than 12 credits)
- ❑ Unemployed
- ❑ Retired
- ❑ Disabled
- ❑ Other: ______________________

10. If you are working, where do you work? ____

11. If you are working, what is your current job title?
____

12. What is your monthly income?
- ❑ $0 - $1,000 ($0-$12,000 per year)
- ❑ $1,001 - $2,000 ($12,012-$24,000 per year)
- ❑ $2,001 - $3,000 ($24,012-$36,000 per year)
- ❑ $3,001 - $4,000 ($36,012-$48,000 per year)
- ❑ $4,001 - $5,000 ($48,000-$60,000 per year)
- ❑ $5,001+ (over $60,000 per year)

13. What is your marital status?
- ❑ Now married
- ❑ Widowed
- ❑ Divorced
- ❑ Separated
- ❑ Never married

14. Who do you live with? (check all that apply)
- ❑ Alone
- ❑ Just partner
- ❑ Just child/children
- ❑ Partner and child/children
- ❑ Extended or blended family
- ❑ Friends
- ❑ Other____

15. When did you find out you have kidney disease? Month ______ Year _______

16. On what date did you begin dialysis? Month ______ Year _______

17. Have you had a gap(s)since the date above where you did NOT receive dialysis?
- ❑ Yes: ____________________________ (Date(s) of gap)
- ❑ No

18. Have you received a kidney transplant?
- ❑ Yes: ____________________________(Date(s) of transplant(s))

- ❑ No (skip question 19)

19. Is your kidney transplant still working?
- ❑ Yes
- ❑ No

20. Apart from Dialysis, do you do anything else to take care of your kidney disease? (check all that apply)
- ❑ Diet (e.g., eating less salt or potassium)
- ❑ Drinking less water or other fluids
- ❑ Exercise
- ❑ Kidney disease drugs *(e.g., diuretics/water pills, ACE inhibitors, iron therapy, etc.)*
- ❑ Other ____
- ❑ Nothing else
- ❑ Not sure

21. How often do you experience low blood pressure (hypotension, which is also known as "crashing" and can have symptoms such as sweating, nausea, and light headedness) during dialysis treatment?
- ❑ Every session (about 3 times per week)
- ❑ About 2 times per week
- ❑ About 1 time per week
- ❑ 1-3 times a month
- ❑ Every 1-3 months
- ❑ Every 4-6 months
- ❑ Less often than every 6 months, but more often than never
- ❑ Never
- ❑ Not sure

22. Do you currently do volunteer work with any organization?
- ❑ Yes
- ❑ No

23. If you are volunteering, with which organization(s) do you do this?
____

24. If you are volunteering, what volunteer work do you do?____
____

25. Have you ever been a peer mentor for other people with kidney disease?
- ❑ Yes
- ❑ No (skip to question 26)

26. When did you begin peer mentoring? ____ _____ (mm/yyyy)

27. About how many people with kidney disease have you peer mentored? _________

28. About how often do you use the Internet or e-mail from each of these places?

a. Home (check one)
- ❑ Several times a day
- ❑ About once a day
- ❑ 3-5 days a week
- ❑ 1-2 days a week
- ❑ Every few weeks

- ❑ Less often
- ❑ Never
- ❑ Don't know

b. Work (check one)
- ❑ Several times a day
- ❑ About once a day
- ❑ 3-5 days a week
- ❑ 1-2 days a week
- ❑ Every few weeks
- ❑ Less often
- ❑ Never
- ❑ Don't know

c. Public Library (check one)
- ❑ Several times a day
- ❑ About once a day
- ❑ 3-5 days a week
- ❑ 1-2 days a week
- ❑ Every few weeks
- ❑ Less often
- ❑ Never
- ❑ Don't know

d. Mobile Device outside of home, work or library (check one)
- ❑ Several times a day
- ❑ About once a day
- ❑ 3-5 days a week
- ❑ 1-2 days a week
- ❑ Every few weeks
- ❑ Less often
- ❑ Never
- ❑ Don't know

e. Other: _________________ (check one)
- ❑ Several times a day
- ❑ About once a day
- ❑ 3-5 days a week
- ❑ 1-2 days a week
- ❑ Every few weeks
- ❑ Less often
- ❑ Never
- ❑ Don't know

29. When you use the Internet or e-mail, how do you <u>USUALLY</u> do that? (check all that apply)
- ❑ Using a desktop computer
- ❑ Using a laptop computer
- ❑ Using a regular cell phone
- ❑ Using a smart phone or other handheld device (e.g., iPhone, Android, Blackberry, Palm, iPod Touch)
- ❑ Using a tablet computer (eg., iPad, Android tablet, Kindle Fire)
- ❑ Using a game console (e.g., PlayStation, X-Box)
- ❑ Other:_________________
- ❑ Don't know

- ❑ Not applicable

30. When you use the Internet or e-mail, what kind of Internet connection do you USUALLY have? (check all that apply)

- ❑ Cellular data plan for a smartphone or other mobile device
- ❑ Broadband (high-speed) Internet service such as cable, fiber optic, or DSL service
- ❑ Satellite Internet service
- ❑ Dial-up Internet service
- ❑ Other ____
- ❑ Don’t know

31. About how many years have you used the Internet or email? ______ Years

THANK YOU!

Default Question Block

Thank you for your interest in participating in the Dialysafe Research study. On the next page will be a consent form. Please read the consent carefully. If you agree to be in the research study, there will be a survey following the consent form.

Consent to Participate in a Research Study

Dialysafe

Overview and purpose
We are asking you to answer some survey questions as part of a research study called: "Enhancing the cardiovascular safety of hemodialysis care: a cluster-randomized, comparative effectiveness trial of multimodal provider education and patient activation interventions." Findings from the survey will be used to develop interventions to help reduce the occurrence of low blood pressure during dialysis sessions. This study is in partnership with the National Kidney Foundation, Fresenius Medical Care, and the University of Michigan. The principal investigator for this study is Dr. Tiffany Veinot of the University of Michigan.

Description of your involvement
If you agree to participate in this survey, you will be asked questions about your demographic background and your experience with "bad runs" during hemodialysis. We are looking for different points of view, so please say what's on your mind. The survey will take approximately 30-45 minutes to complete.

We would also like to ask for your permission to contact you again in the case that we'd like to get more information on any of your answers in the survey. All participation is voluntary, and you will have the option to say 'yes' or 'no' to participating when we contact you again.

Benefits

While you may not receive a direct benefit from participating, we hope that this study will be beneficial to your community in our effort to make dialysis safer.

Risks and discomforts

You might feel uncomfortable talking about some of the subject matter discussed in the survey.

Confidentiality

Every effort will be made to ensure the confidentiality of the survey records. Only the research team will have access to the records. To further ensure confidentiality, please do not use your real name in your survey responses. Even if a name is mentioned, it will not be included in our research data.

In any sort of report we might publish, we will not include any information that will make it possible to identify an individual. Only summary information, such as gender, race/ethnicity, and educational level will be used. However, it will not be linked to any one person. Your exact words may be used in a group report or publication, but you will not be identified by name or by any other information you provide. The researchers plan to keep this study data for approximately seven years.

Also, if you tell us something in the survey that makes us concerned that you or others may have been harmed or that you might harm another person, we have an ethical obligation to report this concern to the Michigan Department of Human Services.

Voluntary nature of the study

Your participation in the survey is totally voluntary, and you may refuse to answer any question. Should you choose at any time to quit participating, it will not affect you in any way. Your decision whether or not to participate will not affect your current or future relationship with National Kidney Foundation, Fresenius Medical Care, or University of Michigan. If you decide to participate, you are free to withdraw at any time. The

information collected up to the point that you withdraw will be included in our analysis unless you specifically ask that it be removed.

Contact information
If you have questions about this research, you can contact Dr. Tiffany Veinot at the University of Michigan at 734-615-8281 or tveinot@umich.edu.

If you have questions about your rights as a research participant, or wish to obtain information, ask questions or discuss any concerns about this study with someone other than the researcher(s), please contact the University of Michigan Health Sciences and Behavioral Sciences Institutional Review Board, 2800 Plymouth Rd. Building 520, Room 1169, Ann Arbor, MI 48109-2800, (734) 936-0933, or toll free, (866) 936-0933, irbhsbs@umich.edu.

Consent
By signing choosing the option, "I agree to participate in this study", you are agreeing to be in the study and to be audiotaped. We will keep a copy of this consent in our study records. Be sure that we have answered your questions about the study and you understand what you are being asked to do. You may contact the researcher if you think of a question later.

- ◯ I agree to participate in this study.
- ◯ I do not agree to participate in this study.

These questions give use some background information about you.

What is your name?

First Name ☐

Last Name ☐

What is your birth month?

☐ ▼

What is your birth year?

[ ]

What is your gender?

- ○ Male
- ○ Female
- ○ [ ] Other (Please type in box)

What is your race? (Choose all that apply)

- ☐ White
- ☐ Black or African American
- ☐ American Indian or Alaska Native
- ☐ Asian
- ☐ Native Hawaiian or Pacific Islander
- ☐ [ ] Other (Please type in box)

With which Asian ethnicity do you identify? (Choose all that apply)

- ☐ Asian Indian
- ☐ Chinese
- ☐ Filipino
- ☐ Japanese
- ☐ Vietnamese
- ☐ Korean
- ☐ [ ] Other (Please type in box)

With which Native Hawaiian/Pacific Islander ethnicity do you identify? (Choose all that apply)

- ☐ Native Hawaiian
- ☐ Guamanian or Chamorro
- ☐ Samoan
- ☐ ________ Other (Please type in box)

Are you Hispanic, Latino/a, or Spanish origin? (Choose all that apply)

- ☐ No, not of Hispanic, Latino/a, or Spanish origin
- ☐ Yes, Mexican, Mexican American, Chicano/a
- ☐ Yes, Puerto Rican
- ☐ Yes, Cuban
- ☐ Yes, another Hispanic, Latino/a, or Spanish origin

What is the postal code where you live?

____

What is the highest degree or level of school that you have completed?

- ○ Grade 8 or less
- ○ Grades 9 to 12, no diploma
- ○ High school graduate or equivalent (GED)
- ○ Some college
- ○ Associates degree (e.g., AA, AS)
- ○ Bachelors degree (e.g., BA, BS)
- ○ Graduate degree (e.g., MA, MS, PhD)
- ○ Professional degree (e.g., MD, JD)

What is your current employment activity? (check all that apply)

- ☐ Working full-time (30 hours or more)
- ☐ Working part-time (less than 30 hours)
- ☐ Full-time student (12 or more credits)

- [ ] Part-time student (less than 12 credits)
- [ ] Unemployed
- [ ] Retired
- [ ] ________ Other (Please type in box)

Where do you work?

____

What is your current job title?

____

What is your monthly income?

- ○ $0-$1,000 ($0-$12,000 per year)
- ○ $1,001-$2,000 ($12,012-$24,000 per year)
- ○ $2,001-$3,000 ($24,012-$36,000 per year)
- ○ $3,001-$4,000 ($36,012-$48,000 per year)
- ○ $4,001-$5,000 ($48,012-$60,000 per year)
- ○ $5,001+ (over $60,000 per year)

What is your marital status?

- ○ Now Married
- ○ Widowed
- ○ Divorced
- ○ Separated
- ○ Never married

Who do you live with? (Check all that apply)

- [ ] Alone

- [ ] Just partner
- [ ] Just child/children
- [ ] Partner and child/children
- [ ] Extended or blended family
- [ ] Friends
- [ ] ______ Other (Please type in box)

In what month did you find out that you have kidney disease?

[▼]

In what year did you find out you have kidney disease?

[ ]

In what month did you begin dialysis?

[▼]

In what year did you begin dialysis?

[ ]

Have you had a gap(s) since the date above where you did NOT receive dialysis?

- ○ ______ Yes, and the date (s) are:
- ○ No

Have you received a kidney transplant?

- ○ ______ Yes, and the date (mm/dd/yyyy) of transplant was:
- ○ No

Is your kidney transplant still working?

- ◯ Yes
- ◯ No

Apart from Dialysis, do you do anything else to take care of your kidney disease? (check all that apply)

- ☐ Diet (e.g., eating less salt or potassium
- ☐ Drinking less water or other fluids
- ☐ Exercise
- ☐ Kidney disease drugs (e.g., diuretics/water pills, ACE inhibitors, iron therapy, etc.)
- ☐ ______ Other (Please type in box)
- ☐ Nothing else
- ☐ Not sure

During dialysis treatment, about how often do you experience low blood pressure (hypotension, which is also known as "crashing" and can have symptoms such as sweating nausea, and light headedness)?

- ◯ Every session (about 3 times a week)
- ◯ 2 times per week
- ◯ 1 time per week
- ◯ 1-3 times a month
- ◯ Every 1-3 months
- ◯ Every 4-6 months
- ◯ Less often than every 6 months, but more often than never
- ◯ Never
- ◯ Not Sure

Have you ever been a peer mentor for other people with kidney disease?

- ○ Yes
- ○ No

In what month did you begin peer mentoring other people with kidney disease?

[▼]

In what year did you begin peer mentoring other people with kidney disease?

[ ]

About how many people with kidney disease have you peer mentored?

[ ]

When you use the Internet or email, how do you usually do that? (Check all that apply)

- ☐ Using a desktop computer
- ☐ Using a laptop computer
- ☐ Using a regular cell phone
- ☐ Using a smart phone or other handheld device (e.g., iPhone, Android, Nlackberry, Palm, iPod Touch)
- ☐ Using a tablet computer (e.g., iPad, Android tablet, Kindle Fire)
- ☐ Using a game console (e.g., PlayStation, X-Box)
- ☐ [ ] Other:
- ☐ Don't know
- ☐ Not applicable

When you use the Internet or email, what kind of Internet connection do you usually have? (Check all that apply)

- ☐ Cellular data plan for a smartphone or other mobile device
- ☐ Broadband (high-speed) Internet service such as cable, fiber optic, or DSL service

- ☐ Satellite Internet service
- ☐ Dial-up Internet sevice
- ☐ ________ Other:
- ☐ Don't know

Block 1

In your opinion, what makes for a "bad run" at dialysis?

Have you ever had a "bad run" at dialysis?

- ◯ Yes
- ◯ No

What happened when you had a "bad run?"

What did the "bad run" feel like?

When you had a "bad run," what did you do in response?

Did anyone help you during or after your "bad run?"

- Yes
- No

What did the person that helped you do?

Why do you think that this "bad run" happened?

Powered by Qualtrics
